# The solution structure of an anti-CRISPR protein

**DOI:** 10.1038/ncomms13134

**Published:** 2016-10-11

**Authors:** Karen L. Maxwell, Bianca Garcia, Joseph Bondy-Denomy, Diane Bona, Yurima Hidalgo-Reyes, Alan R. Davidson

**Affiliations:** 1 Donnelly Centre for Cellular and Biomolecular Research, University of Toronto, Toronto, Ontario, Canada M5S 3E1; 2 Department of Biochemistry, University of Toronto, Toronto, Ontario, Canada M5S 1A8; 3 Department of Molecular Genetics, University of Toronto, Toronto, Ontario, Canada M5S 1A8

## Abstract

Bacterial CRISPR–Cas adaptive immune systems use small guide RNAs to protect against phage infection and invasion by foreign genetic elements. We previously demonstrated that a group of *Pseudomonas aeruginosa* phages encode anti-CRISPR proteins that inactivate the type I-F and I-E CRISPR–Cas systems using distinct mechanisms. Here, we present the three-dimensional structure of an anti-CRISPR protein and map a functional surface that is critical for its potent inhibitory activity. The interaction of the anti-CRISPR protein with the CRISPR–Cas complex through this functional surface is proposed to prevent the binding of target DNA.

## Abstract (web summary)

Recently, anti-CRISPR proteins have been identified. Here, the authors report the solution structure of one of these proteins, and use mutational analysis to provide some insight into its function.

## Graphical abstract

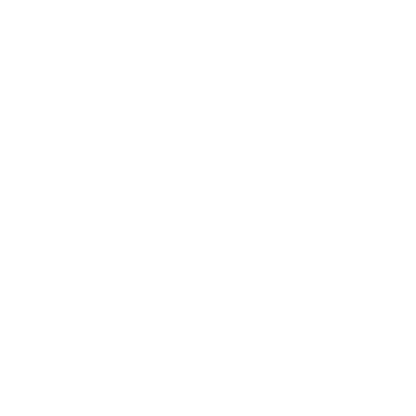

Phage predation is a major selective pressure on bacterial populations, and bacteria have evolved an adaptive immune system, known as CRISPR–Cas, to defend against them. CRISPR loci are comprised of palindromic repeat sequences interspersed with unique short spacer regions that are often identical to phage and plasmid deoxyribonucleic acid (DNA)^1^,^2^,^3^. After transcription, CRISPR ribonucleic acid (RNA) is processed and complexed with Cas proteins to form a genome surveillance system guided by the sequence of the spacer RNA. Sequence-specific binding by this complex to an invading phage genome targets it for degradation. We previously showed that some phages evade CRISPR–Cas-mediated destruction by encoding ‘anti-CRISPR' proteins that inhibit these systems^4^,^5^. One of these proteins, AcrF1, is a potent inhibitor of the type I-F CRISPR–Cas system of *P. aeruginosa*. AcrF1 inhibits the DNA-binding activity of the surveillance complex of this system (called the Csy complex) by interacting with the Csy3 subunit^6^.

In this study, we determine the nuclear magnetic resonance (NMR) solution structure of the anti-CRISPR protein AcrF1. Using site-directed mutagenesis, we identify a functional surface through which it mediates its anti-CRISPR activity. The inactivation of the type I-F CRISPR–Cas system by AcrF1 was found to be dependent on very tight binding to the Csy complex. We believe this strong interaction locks the Csy complex in a conformation that is unable to bind CRISPR-targeted DNA.

## Results

### The structure of AcrF1 reveals a functional interface

**Figure 1:**
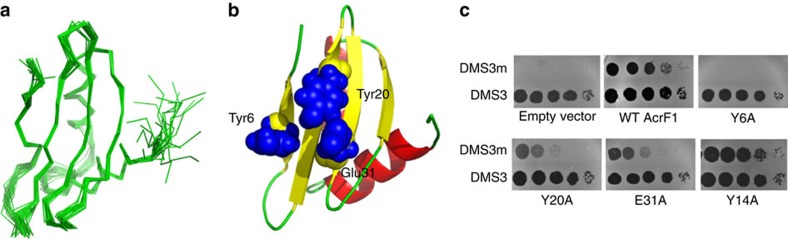
The structure of AcrF1 allowed the identification of a functional interface. (**a**) The NMR solution structure of AcrF1. Overlay of 20 lowest energy AcrF1 structures showing the best-fit superposition of the backbone atoms (N, C^α^ and C′). (**b**) Ribbon representation of AcrF1 showing the positions of the three residues that are critical for AcrF1 function *in vivo*. (**c**) Tenfold serial dilutions of phage lysates with a starting concentration of 10^7^ pfu mL^−1^ were spotted onto a lawn of bacteria containing an active type I-F CRISPR–Cas system. Replication of CRISPR-sensitive phage DMS3m is inhibited unless a fully functional AcrF1 anti-CRISPR is expressed from a plasmid within the cells. Phage DMS3 is not targeted by the CRISPR–Cas system. Phage replication results in round zones of clearing of the bacterial lawn. Y14A is a fully active mutant shown for comparison.

To gain mechanistic insight into the function of AcrF1 we solved its three-dimensional (3D) structure by NMR spectroscopy. A combination of two-dimensional and 3D experiments were used to identify and sequentially assign greater than 95% of the ^1^H, ^15^N and ^13^C resonances of the backbone and side chain atoms. A total of 1,029 distance and 51 dihedral angle restraints were used in the structure determination. The ensemble of 20 low-energy structures calculated for AcrF1 is presented in Fig. 1a, and the statistics of the structure calculations in Table 1. The structure of AcrF1 is composed of four anti-parallel β-strands forming a single β-sheet and two anti-parallel α-helices (Fig. 1b). The α-helices pack against one side of the β-sheet, forming a tightly packed hydrophobic core with 13 residues >90% buried. A DALI search^7^ to identify proteins with similar 3D folds in the Protein Data Bank revealed that AcrF1 lacked informative structural similarity to other proteins.

**Figure 2:**
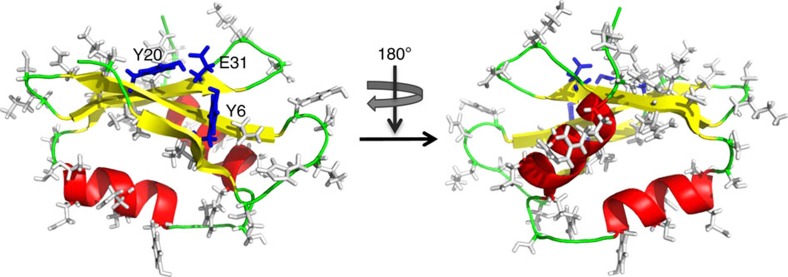
Alanine scanning mutagenesis of AcrF1. The side chains of substituted surface-exposed residues are indicated. Substitution of the side chains shown in grey had a 10-fold or less effect on *in vivo* anti-CRISPR activity. Substitution of the three residues shown in blue resulted in a 100-fold or greater decrease in *in vivo* activity.

To identify the surfaces of AcrF1 that mediate anti-CRISPR activity, we individually substituted 35 surface-exposed residues that were widely distributed across the structure with alanine (Fig. 2, Table 2). The *in vivo* anti-CRISPR activity of each mutant was assayed by measuring its capacity to enable phage replication in a *P. aeruginosa* strain bearing an active CRISPR–Cas system (Fig. 1c). Of the 35 mutants created, one (Y6A) displayed a 10^7^-fold decrease in *in vivo* anti-CRISPR activity, and two others (Y20A and E31A) displayed ∼100-fold reductions in activity. The remaining mutants showed changes in activity of 10-fold or less. Additional substitutions at Tyr6 revealed less severe phenotypes than observed for Y6A, with a 100-fold decrease in activity for Y6N and 10-fold decrease for Y6H. The reason why these mutants do not inhibit activity to the same extent as Y6A is not clear. Circular dichroism spectroscopy of the three alanine mutants with reduced activity revealed spectra that were similar, but not identical to the wild-type protein. Their cooperative temperature-induced unfolding curves and melting temperatures near wild type indicated that these mutants maintained folded structures (Supplementary Fig. 1). Since the Tyr6, Tyr20 and Glu31 residues are clustered in a single patch on the surface of the AcrF1 β-sheet (Fig. 1b), we conclude that this region comprises a critical functional interface required for anti-CRISPR activity.

### Tight binding of AcrF1 is required for activity

**Figure 3:**
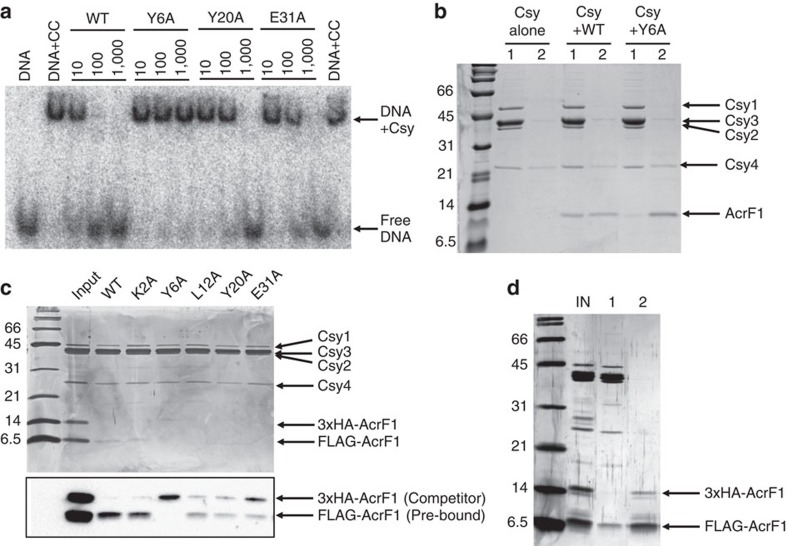
AcrF1 *in vitro* binding strength correlates with *in vivo* activity. (**a**) EMSA was used to assay binding of the Csy complex to target DNA in the presence of wild-type or mutant AcrF1 proteins. The anti-CRISPR proteins were added to the Csy complex in 10-, 100- or 1,000-fold excess. The ‘DNA+CC' lanes contain DNA and Csy complex with no AcrF1. (**b**) Mixtures of Csy complex and AcrF1wild type or Y6A mutant were fractionated by size exclusion chromatography. The lanes show (1) peak fractions for the Csy complex with or without AcrF1 bound and (2) peak fractions for unbound AcrF1. (**c**) The indicated AcrF1-FLAG mutant proteins were pre-mixed with the Csy complex, and then competed by addition of wild-type AcrF1-HA. Csy complexes were isolated by size exclusion chromatography and the peak fraction from each sample was analysed by SDS–PAGE followed by silver staining. Western blots of the same gel, shown below, were used to identify the differentially tagged AcrF1 proteins. (**d**) Wild-type AcrF1-FLAG was incubated with Csy complex and then competed with wild-type AcrF1-3xHA for 16 h. Size exclusion chromatography was used to separate the AcrF1 bound to the Csy complex (1) from free AcrF1 (2).

As our previous studies showed that AcrF1 blocked DNA binding by the Csy complex^6^, we examined the ability of the most severely compromised mutant to inhibit this activity. Electrophoretic mobility shift assays showed that the AcrF1(Y6A) mutant was unable to block binding of the Csy complex to a 50-nucleotide double-stranded DNA target containing a protospacer and a protospacer adjacent motif even when present in 1,000-fold excess (Fig. 3a). By contrast, the wild-type protein inhibited DNA binding when present in 10-fold excess of the Csy complex, and completely blocked binding at 100-fold excess. The two AcrF1 mutants that showed intermediate reductions in *in vivo* activity (Y20A and E31A) blocked DNA binding when present at 1,000-fold excess, but not at 100-fold excess (Fig. 3a). Thus, the level of *in vivo* activity of AcrF1 mutants correlates with their ability to block the targeted DNA binding of the Csy complex *in vitro*, with partially active mutants evincing a partial inhibition of DNA-binding activity.

After showing that the most severely compromised mutant anti-CRISPR protein, AcrF1(Y6A), was unable to block DNA binding, we next assessed its ability to bind the Csy complex (Supplementary Fig. 2). Purified *P. aeruginosa* Csy complex was mixed *in vitro* with a 10-fold excess of wild type or Y6A mutant protein, and the complexes were purified using size exclusion chromatography. While the Y6A mutant was able to bind the Csy complex (Fig. 3b), much less co-eluted with the Csy complex as compared with wild-type AcrF1, suggesting a lower binding affinity.

To further characterize the binding of mutant AcrF1 proteins to the Csy complex, we performed competition experiments. FLAG-tagged mutant AcrF1 was pre-bound to Csy complex that was immobilized on beads. Subsequently, HA-tagged wild-type AcrF1 was added as a competitor. When the inactive Y6A mutant was tested, it was fully displaced by wild-type AcrF1 after 1 h (Fig. 3c). By contrast, when wild-type FLAG-AcrF1 was challenged with wild-type HA-AcrF1, no dissociation was observed after 1 h (Fig. 3c). In fact, wild-type FLAG-AcrF1 did not exchange with HA-AcrF1 even after a 16 h incubation (Fig. 3d), illustrating its very tight binding to the Csy complex. An alanine mutant with wild-type anti-CRISPR activity *in vivo* (K2A) was also not significantly displaced by wild-type AcrF1, while the Y20A and E31A mutants showed ∼50% exchange at 1 h when competed by wild-type protein (Fig. 3c). These experiments show that the *in vivo* activities of the AcrF1 mutants also correlate with their binding affinity to the Csy complex with the Y20A and E31A mutants binding more weakly than wild type, but more strongly than the Y6A mutant. Thus, we conclude that AcrF1 inhibition of the DNA binding activity and *in vivo* function of the Csy complex is dependent on its ability to bind tightly to the Csy complex.

## Discussion

In this work, we determined the first structure of an anti-CRISPR protein and provide insight into the interactions that mediate its activity. The ability of AcrF1 to profoundly inhibit the activity of the *P. aeruginosa* type I-F CRISPR system is mediated through a single interface on one surface of the protein. Our previous work showed that two or three molecules of AcrF1 bind to the Csy complex at a site distinct from the DNA-binding interface, suggesting an allosteric mechanism for inhibition of DNA binding^6^. Here we show that AcrF1 binds very tightly to the Csy complex with no dissociation observed at 16 h, and that strong binding is a requirement for full biological activity. This potent interaction by AcrF1 likely locks the Csy complex into a conformation that is incompatible with DNA binding. Further elucidation of the conformational plasticity inherent in CRISPR–Cas complexes, as will be provided by investigation of their inhibitors, will supply crucial new insights into the functioning of these important systems.

## Methods

### NMR spectroscopy

AcrF1 was expressed from a p15TV-L plasmid (Novagen) with an N terminal, tobacco etch virus (TEV)-cleavable 6-His tag. *Escherichia coli* strain ER2566 cells harbouring this plasmid were grown at 37 °C in M9 minimal medium enriched with 2.5 g l^−1^ of [^13^C] glucose, 0.7 g L^−1^ [^15^N] NH_4_Cl to an OD_600_ of 1.0, and protein expression was induced by the addition of 175 μg ml^−1^ isopropyl β-D-1-thiogalactopyranoside (IPTG). The cells were incubated at 37 °C for an additional 3 h, then were collected by centrifugation and AcrF1 was purified as previously described^6^. The 6-His tag was cleaved by incubation overnight with 6-His tagged TEV protease. The sample was passed over Ni-nitrilotriacetic acid (NTA) resin (Qiagen) to remove the TEV protease, the cleaved tag, and any uncleaved AcrF1 protein. Size exclusion chromatography on a 25 ml Superdex-75 column (Pharmacia) was used as a final purification step.

Spectra were collected with 1 mM protein in 25 mM Na_2_HPO_4_ (pH 6.8), 200 mM NaCl and a ^2^H_2_O concentration of 10% (all spectra) or 100% (^13^C-NOESY). NMR data were obtained on a Bruker 800-MHz at 25 °C at the Quebec/Eastern Canada High Field NMR Facility. NMR data processing and analysis was performed using NMRPipe^8^, and spectra were analysed with Sparky^9^. Structure calculations were performed by CYANA 3.0 (ref. 10) using automatically assigned and manually verified distance restraints from ^15^N- and ^13^C-NOESY experiments, and dihedral angle restraints derived by TALOS (ref. 11). Fifty structures were calculated, and the 20 lowest energy structures were selected and analysed. Structural quality was assessed by PROCHECK (ref. 12). The structure ensemble was deposited in the Protein Data Bank under the code 2LW5, and the chemical shifts were deposited in the Biological Magnetic Resonance Databank under entry number 18606.

### AcrF1 mutagenesis

Mutations were introduced by site-directed mutagenesis into the AcrF1 open reading frame, contained in either the pHERD30T plasmid (for anti-CRISPR activity assays in *P. aeruginosa*) or the p15TV-L plasmid (for protein expression and purification in *E. coli*). The solvent accessible residues were determined using Swiss-PbdViewer^13^. The pHERD30T plasmids were electroporated into *P. aeruginosa* strain PA14 to assay anti-CRISPR activity, and the p15TV-L plasmids were introduced into *E. coli* ER2566 for protein purification.

### *In vitro* protein competition assays

Purified Csy complex^6^ was pre-incubated for 1 h with a 10-fold molar excess of purified FLAG-tagged mutant or wild-type AcrF1 protein to allow binding to occur. Equal amounts of HA-tagged wild-type AcrF1 protein were then added to the sample and allowed to incubate in competition for 1 h. Different tags were present on the two proteins to ensure that they would migrate to different positions on an SDS–polyacrylamide gel electrophoresis (PAGE) gel. The mixture was fractionated by size exclusion chromatography to separate the Csy complex and bound proteins. The size exclusion fractions corresponding to the peak containing the co-eluting AcrF1/Csy complex and the peak of the AcrF1 protein alone were analysed by SDS–PAGE followed by silver staining or Western blotting using anti-His (BioShop; TA6001), anti-FLAG (MB Biomedicals; 68720) or anti-HA (Santa Cruz Biotech; SC7392) antibodies. Each experiment was completed three times.

### Plaque assays

To assess the activity of various AcrF1 mutants *in vivo*, the pHERD30T plasmid was used to transform *P. aeruginosa* strain PA14. The PA14 CRISPR–Cas system naturally targets phage DMS3*m*, preventing its replication, while phage DMS3 is insensitive to the CRISPR–Cas system. Phage lysates at a starting concentration of 10^7^ plaque-forming units per mL were spotted in 10-fold serial dilutions onto a lawn of PA14 containing empty vector, wild-type AcrF1, or mutant AcrF1 and plaquing efficiencies at 30 °C were determined for three separate experiments.

### Electrophoretic mobility shift assays

A 50 bp double-stranded DNA molecule was generated through the synthesis of two complementary single-stranded DNA molecules (Eurofins Genomics) that were annealed together by heating to 98 °C and cooling gradually to room temperature. One strand had 32 nucleotides of complementarity with the crRNA in the purified Csy complex and was labelled at the 5′ end with [^32^P]. The electrophoretic mobility shift assay (EMSA) experiment was conducted in binding buffer (10 mM HEPES, pH 7.5, 1 mM MgCl_2_, 20 mM KCl, 1 mM TCEP, bromophenol blue and 6% glycerol) at 37 °C for 15 min. The Csy complex was routinely used at a concentration of 100 nM in reactions, with 1 nM labelled DNA. Wild-type AcrF1 or the indicated protein variants were used at molar excess compared with the Csy complex as indicated in the relevant figures. AcrF1 was incubated with the Csy complex for 1 h at 4 °C. After the appropriate incubation, the reactions were resolved on native 6% polyacrylamide Tris-Borate-EDTA (TBE) gels. Gels were wrapped in Saran wrap and visualized with a phosphoscreen on a Typhoon imager (GE Healthcare Life Sciences).

### Data availability

AcrF1 structure coordinates have been deposited in the Protein Data Bank with the accession code 2LW5 (http://www.rcsb.org/pdb/explore.do?structureId=2lw5). The protein chemical shift data was deposited in the Biological Magnetic Resonance Databank under entry number 18606 (http://bmrb.cerm.unifi.it/data_library/summary/index.php?bmrbId=18606).

## Additional information

**How to cite this article:** Maxwell, K. L. *et al*. The solution structure of an anti-CRISPR protein. *Nat. Commun.* **7,** 13134 doi: 10.1038/ncomms13134 (2016).

## Supplementary Material

Supplementary Information Supplementary Figures 1-2.

## Figures and Tables

**Table 1:** Structural statistics for the ensemble of 20 low-energy structures of AcrF1.

	**Number of restraints**
Distance restraints*	
Short range, |*i−j*|≤1	592
Medium range, 1<|*i−j*|<5	153
Long range, |*i−j*|≥5	384
*Dihedral angle restraints*	
*ϕ*, *ψ* pairs	51
Pairwise r.m.s.d. (Å)†	
Backbone atoms	0.3
All heavy atoms	0.9
*Ramachandran statistics (%)*‡	
Most favored regions	81.2
Additional allowed regions	17.8
Generously allowed regions	0.1
Disallowed regions	0.0

^*^There were no distance violations >0.5 A.

^†^r.m.s.d. for ordered residues; 2–8, 15–22, 24–54, 57–59, 63–73.

^‡^Determined using PROCHECK-NMR (ref. 12).

**Table 2:**
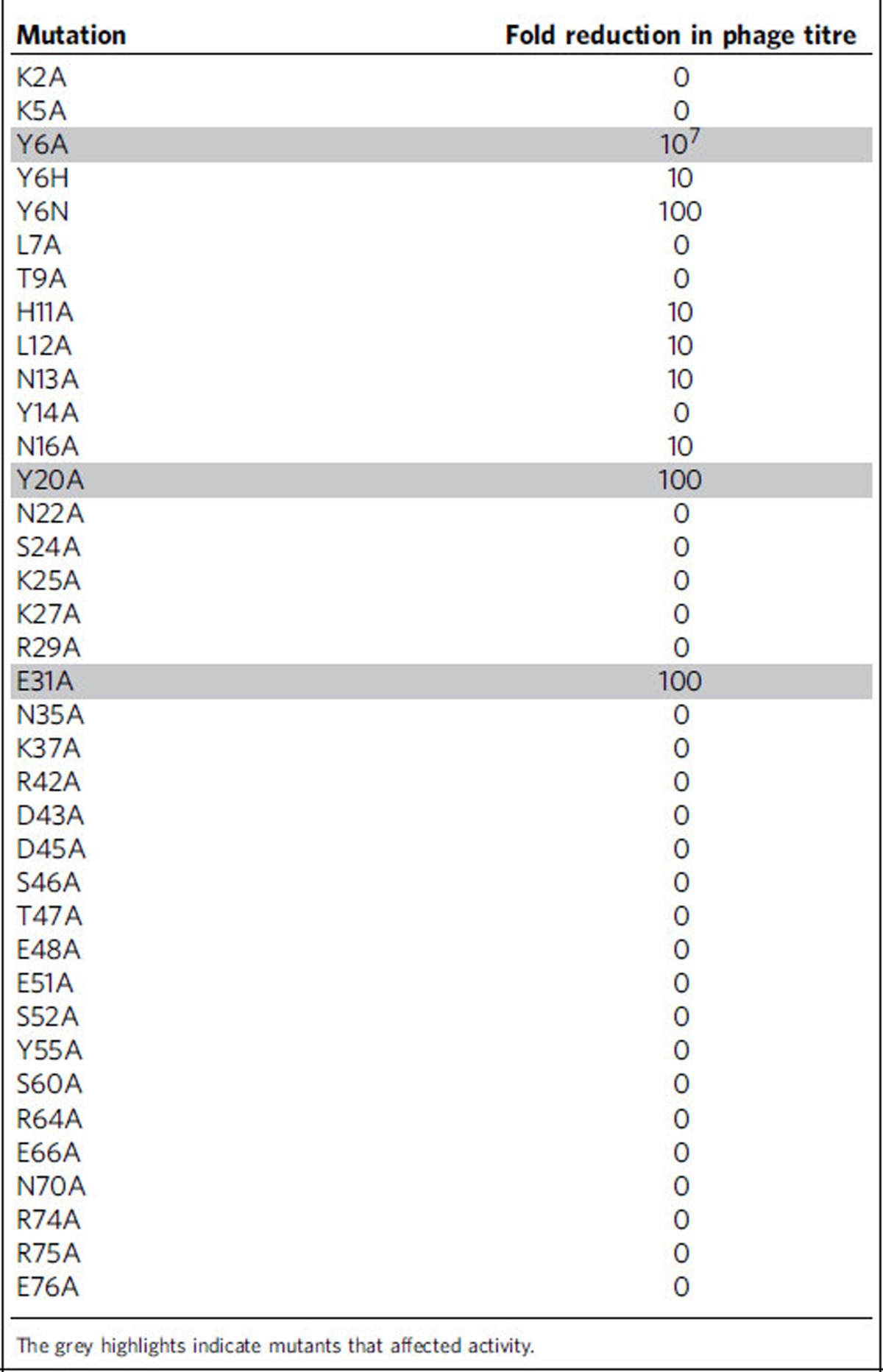
Summary of surface mutations made to AcrF1.
