# Supplementary material for: The solution structure of an anti-CRISPR protein
Source: Nat Commun. 2016 Oct 11;7:13134. doi: 10.1038/ncomms13134 (PMC5062604; doi:10.1038/ncomms13134)
Supplement: Supplementary Information — Supplementary Figures 1-2. [file ncomms13134-s1.pdf]

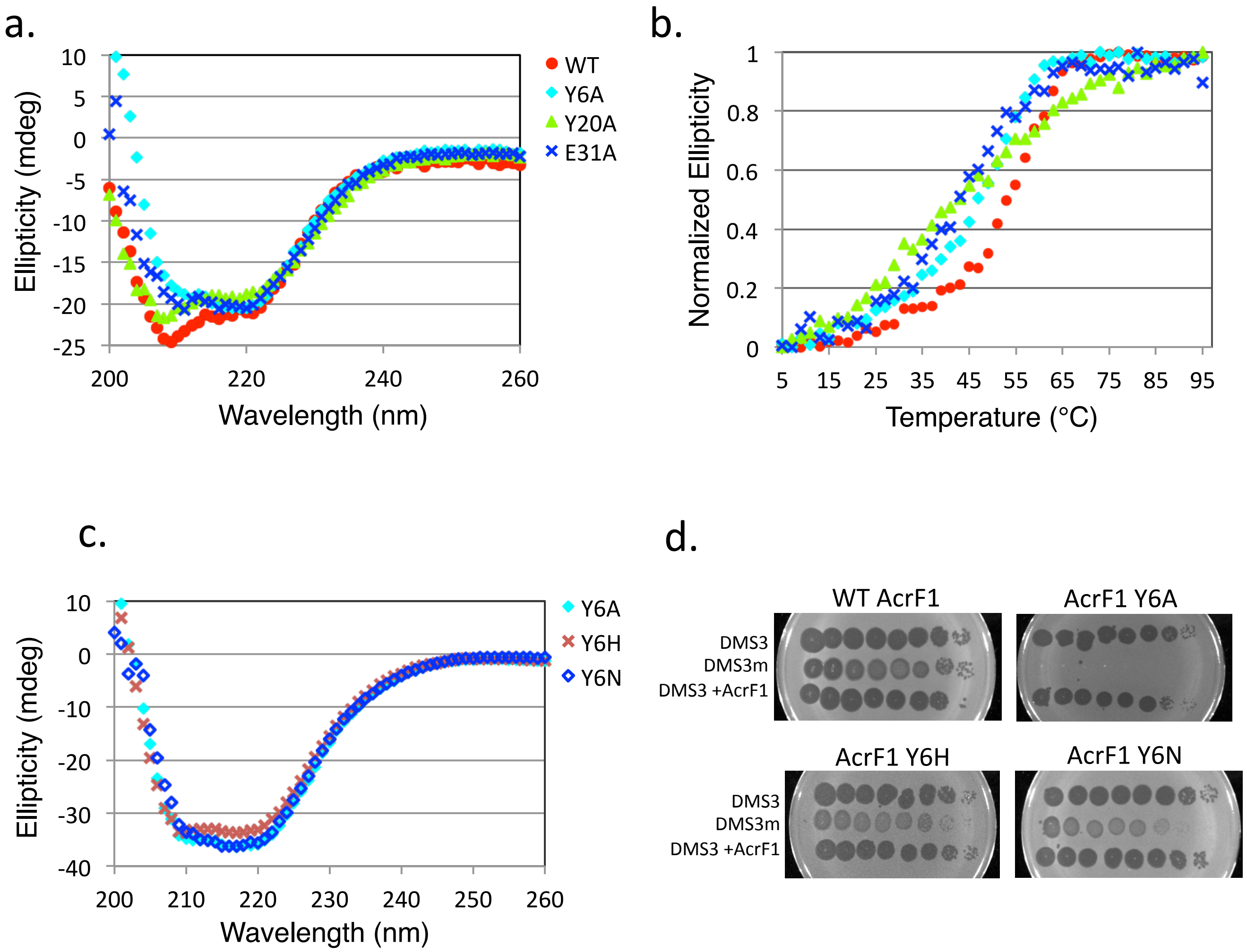

Supplementary Figure 1. Circular dichroism spectroscopy shows that the three AcrF1 mutants that show decreased activity *in vivo* have folded spectra that are similar to the wild type protein (**a**) and maintain wild type stability as assessed by temperature-induced unfolding curves (**b**). While the shape of the CD spectrum for the Y6A mutant is different than that of the wild type protein, this does not appear to be due to large structural rearrangements of the protein that completely abrogate biological activity. Other substitutions at position Y6 that also display altered CD spectra (**c**) led to a 10-fold (Y6H) and 100-fold (Y6N) decrease in activity instead of complete inhibition like Y6A (**d**).

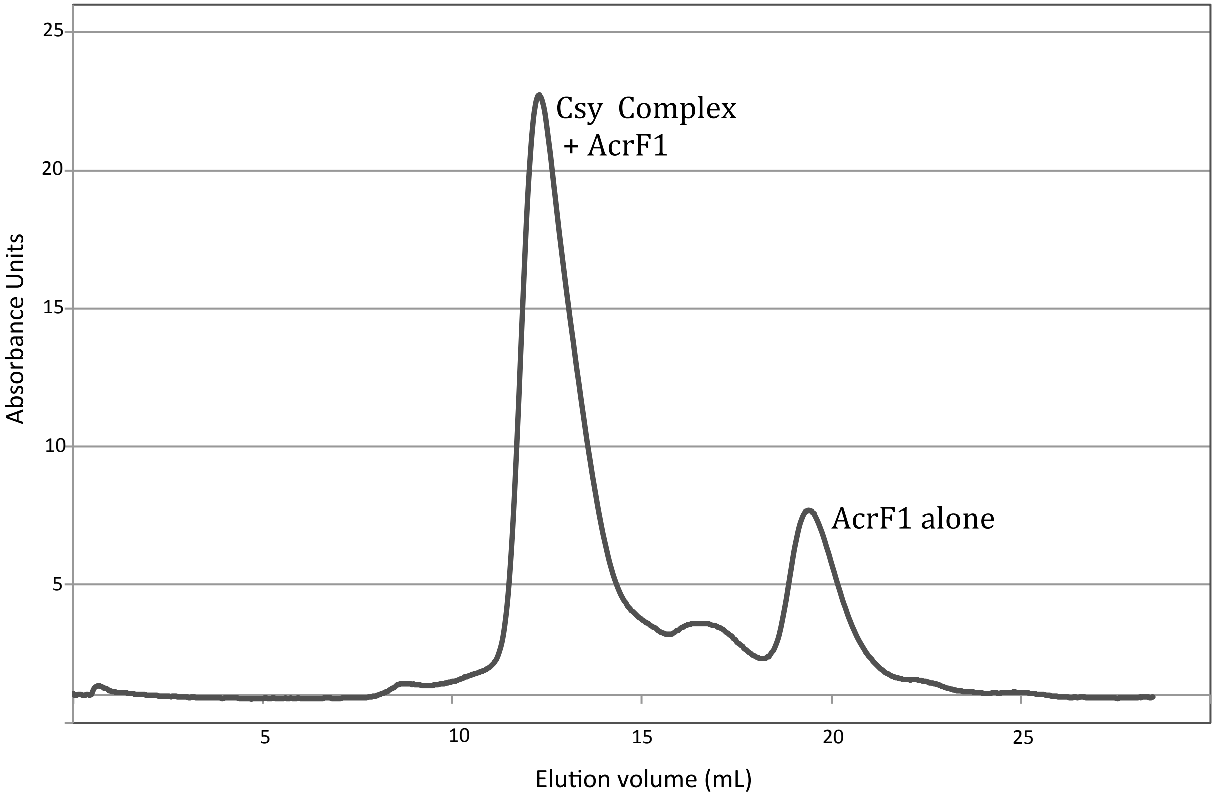

Supplementary Figure 2. Size exclusion chromatography is able to efficiently resolve free AcrF1 from AcrF1 that is bound to the Csy complex.
